# Efficient Transduction of Feline Neural Progenitor Cells for Delivery of Glial Cell Line-Derived Neurotrophic Factor Using a Feline Immunodeficiency Virus-Based Lentiviral Construct

**DOI:** 10.1155/2011/378965

**Published:** 2010-09-20

**Authors:** X. Joann You, Ping Gu, Jinmei Wang, Tianran Song, Jing Yang, Chee Gee Liew, Henry Klassen

**Affiliations:** ^1^Department of Ophthalmology, School of Medicine, Gavin Herbert Eye Institute, University of California, Irvine, 101 The City Drive, Bldg. 55, 2nd Fl, Orange, CA 92868-4380, USA; ^2^Department of Ophthalmology, Shanghai Ninth People's Hospital, School of Medicine, Shanghai Jiaotong University, Shanghai 200011, China; ^3^Department of Cell Biology and Neuroscience, Stem Cell Center, University of California, Riverside, Riverside, CA 92521, USA

## Abstract

Work has shown that stem cell transplantation can rescue or replace neurons in models of retinal degenerative disease. Neural progenitor cells (NPCs) modified to overexpress neurotrophic factors are one means of providing sustained delivery of therapeutic gene products in vivo. To develop a nonrodent animal model of this therapeutic strategy, we previously derived NPCs from the fetal cat brain (cNPCs). Here we use bicistronic feline lentiviral vectors to transduce cNPCs with glial cell-derived neurotrophic factor (GDNF) together with a GFP reporter gene. Transduction efficacy is assessed, together with transgene expression level and stability during induction of cellular differentiation, together with the influence of GDNF transduction on growth and gene expression profile. We show that GDNF overexpressing cNPCs expand in vitro, coexpress GFP, and secrete high levels of GDNF protein—before and after differentiation—all qualities advantageous for use as a cell-based approach in feline models of neural degenerative disease.

## 1. Introduction

The retina is susceptible to a variety of degenerative diseases, including age-related macular degeneration (AMD), retinitis pigmentosa (RP) and other inherited photoreceptor degenerations, photoreceptor loss following retinal detachment, ganglion cell loss in glaucoma and optic neuropathies, as well as the loss of retinal neurons associated with nondegenerative conditions such as diabetic retinopathy (DR), macular edema and ischemia, vascular occlusions, trauma, and inflammatory diseases. Any of these can lead to debilitating visual deficits. AMD is a particularly prevalent cause of blindness among elderly persons, affecting more than 30 million people globally. That number is expected to double over the next decade in association with demographic shifts towards an older population, particularly in developed countries [1]. Similar to the situation with many neurological diseases, little is available in the way of effective treatments for patients with AMD or other blinding disorders of the retina.

A large body of research has shown that the use of exogenous neurotrophic factors can reproducibly promote the survival of specific neurons in various parts of the central nervous system (CNS), including the retina [2, 3]. Frequently investigated neuroprotective neurotrophic factors have included glial cell line-derived neurotrophic factor (GDNF), brain-derived neurotrophic factor (BDNF), and ciliary neurotrophic factor (CNTF). Among these, GDNF has been associated with significant effects with respect to preventing cell death [4], including the protection of specific neuronal populations in the brain [5, 6], spinal cord [7], and retina [8–11]. Receptors for GDNF are known to be expressed within the mature retina [8, 11, 12].

Stem and progenitor cell transplantation has also shown considerable promise in animal models of neural degeneration. Subretinal transplantation of neural progenitor cells (NPCs) has yielded intriguing evidence of cellular repopulation of damaged retinas, growth of neurites into the optic nerve head and retardation of ongoing retinal degeneration [13–17]. Both unmodified, as well as genetically modified, cortical human NPCs can survive for prolonged periods, migrate extensively, secrete growth factors, and rescue visual function following subretinal transplantation in the dystrophic Royal College of Surgeons rat [18], with sustained visual benefits following injection [19]. More recently, subretinal transplantation of human forebrain progenitor cells has been extended to nonhuman primates [20], although this model used nondystrophic hosts and therefore did not lend itself to evaluation of neuroprotective efficacy. When used for transplantation therapy, NPCs engineered to secrete GDNF contributed to reduced apoptotic death in vitro, enhanced survival in vivo, neuronal differentiation, and improved host cognitive function following traumatic brain injury as compared with nontransduced NPCs [21–24]. 

The visual system of the cat is quite sophisticated and one of the most extensively studied among higher mammals. There are many similarities to the human retina although that of the cat has a tapetum and is generally optimized for performance under scotopic conditions [25]. Like humans, the cat is a species with a robust intraretinal circulation [26]. The cat retina has also been the subject of decades of anatomical and physiological studies and has been used as an animal model of binocular visual function as well as studies involving drug treatment and research on retina detachment [27, 28]. In addition, the feline eye is large relative to that of rodents thereby allowing the application of surgical techniques similar to those typically used clinically. Finally, there exist feline models of retinal degeneration caused by spontaneous mutations in genes known to be involved in retinitis pigmentosa in humans [29, 30]. These animals provide excellent models for exploring the therapeutic potential of stem cell-based neuroprotective strategies in an animal with highly developed visual capabilities. 

Previously, we showed that it is possible to derive NPCs from the developing cat brain and that these cells are capable of integration into the retina of dystrophic feline recipients [23]. To more fully exploit the potential of this model, it is useful to develop feline NPCs capable of sustained growth factor delivery to the host retina. Here we use a bicistronic feline lentiviral vector to generate genetically modified feline neural progenitor cells that exhibit sustained overexpression of GDNF before and after differentiation.

## 2. Materials and Methods

### 2.1. Isolation and Culture of Neural Progenitor Cells from Feline Brain

Cat neural progenitor cells (cNPCs) were originally isolated from 47 day cat fetuses as previously described [23]. Briefly, forebrains were removed and finely minced with a surgical scalpel and the resulting tissue fragments digested for 20 minutes in 0.1% type I collagenase (Invitrogen, Carlsbad, CA). The supernatant containing dissociated cells was then passed through a 100 mm mesh strainer, centrifuged, and seeded in complete culture medium, designated here as standard medium (SM), consisting of advanced DMEM/F12, 1% N2 neural supplement, 2 mM L-glutamine, 50 mg/mL penicillin-streptomycin, and epidermal and basic fibroblast growth factors (recombinant human EGF and bFGF, Invitrogen), both at final concentrations of 20 ng/mL. After initial isolation, all medium was changed to an Ultraculture-based composition, identical to the above but in which DMEM/F12 was replaced with Ultraculture serum-free medium (Lonza, Basel, Switzerland). Therefore, in the present study standard proliferation medium was Ultraculture-based with growth factors and is designated (UM), whereas differentiation medium was Ultraculture-based as well, but did not contain added growth factors and did include 10% fetal bovine serum (UM-FBS). Culture medium was changed every 2 days and proliferating cells passaged at regular intervals of 4-5 days.

### 2.2. Lentivector Production and Titer Determination

The lentiviral vector used in this study was an FIV-based bicistronic vector (GeneCopoeia, Germantown, Maryland) designated as lenti-GDNF-GFP, which carries a human GDNF gene driven by the cytomegalovirus (CMV) immediate-early promoter as well as an enhanced green fluorescent protein (GFP) reporter gene with an internal ribosome entry site (IRES). Lenti-GDNF-GFP vectors were prepared by transient transfection of 293T cells using a standard calcium phosphate precipitation protocol (Clontech, Mountain View, CA). Briefly, 293T cells cultured in 10 cm tissue culture dishes (BD Biosciences, San Jose, CA) were transfected with 2 *μ*g of lentiviral transfer vector plasmid, along with 10 *μ*g of the mixed envelope and packaging plasmids. The viral supernatants were harvested 48 and 72 hours posttransfection and concentrated by centrifugation of virus-containing supernatant through a Centricon Plus-70 filter (Millipore, Billerica, MA) following the manufacturer's instruction. Titers of the concentrated lentivector were estimated by transducing cNPC cells with a serial dilution of the virus and flow cytometric identification of GFP-positive cells.

### 2.3. Lentiviral Vector Transduction

Cat neural progenitor cells were transduced with lenti-GDNF-GFP vectors at a MOI of 10 following the standard procedure. Briefly, cNPCs were seeded at a density that allowed them to grow to 90% confluency on the day of transduction. The cells were then transduced by 6–24 hours of exposure to virus-containing supernatant in the presence of 5–8 *μ*g/mL polybrene. Viral vector-containing medium was then replaced with fresh medium and cells were incubated at 37°C in a CO_2_ incubator.

### 2.4. FACS Analysis and Selection of Lenti-GDNF-GFP Positive cNPCs

Cells were harvested using TrypLE Express (Invitrogen) and filtered through cell strainer caps (35 *μ*m mesh) to obtain a single cell suspension (approximately 10^6^ cells per mL for analysis, 0.5–2 × 10^7^ cells per mL for sorting). The stained cells were analyzed and sorted on a fluorescence-activated cell sorter FACSAria (BD Biosciences) using FACSDiva software (BD Biosciences). The fluorochromes were excited by the instrument's standard 488 nm and 633 nm lasers, and green fluorescence was detected using 490 LP and 510/20 filters. Prior to sorting, the nozzle, sheath, and sample lines were sterilized with 70% ethanol or 2% hydrogen peroxide for 15 minutes, followed by washes with sterile water. A 100 *μ*m ceramic nozzle (BD Biosciences), sheath pressure of 20–25 pounds per square inch (PSI), and an acquisition rate of 1,000–3,000 events per second were used as conditions previously optimized for neuronal cell sorting.

### 2.5. Cell Growth Assessment

The growth properties of transduced and nontransduced cNPCs were assessed by culturing both types of cells under proliferation conditions in Ultraculture-based medium (UM). Cells of identical passage number (p17) were seeded in four T25 culture flasks at a density of 0.25 million cells/flask. One flask of each cell type were trypsinized and counted daily. Cell numbers were graphed at each time point to compare the growth properties of transduced versus nontransduced cells.

### 2.6. ELISA Analysis

Transduced and nontransduced cNPCs of identical passage number were seeded in T25 culture flasks (0.25 million/flask). Following attachment of cells (approx. 4 hours), the original media were replaced with 3 mL of fresh media. Subsequently, 3 mL of conditioned media were collected and replaced with fresh media at 24 hour intervals and conditioned samples were saved at −80°C for ELISA analysis. ELISA was performed using a human GDNF DuoSet ELISA kit and protocol (R&D Systems, Minneapolis, MN). Wells of microtiter plates were coated (overnight, room temperature) with 2 *μ*g/mL of GDNF capture antibody in 100 *μ*L of coating buffer (0.05 M Na_2_CO_3_, 0.05 M NaHCO_3_, pH 9.6) and then blocked with 0.1% BSA in PBS for 1 hour at room temperature. Samples (100 *μ*L) were loaded in triplicates and incubated for 2 hours at room temperature, followed by addition of 100 *μ*L antibody detection antibody (0.1 *μ*g/mL) for an additional 2 hours at room temperature. HRP-conjugated streptavidin (1 : 200) in blocking buffer was then added (20 minutes, room temperature) and the reaction visualized by the addition of 100 *μ*L of substrate solution for 20 minutes. The reaction was stopped with 50 *μ*L H_2_SO_4_ and absorbance at 450 nm was measured with reduction at 540 nm using an ELISA plate reader. Plates were washed five times with washing buffer (PBS, pH 7.4, containing 0.05% (v/v) Tween 20) after each step. As a reference for quantification, a standard curve was established by a serial dilution of recombinant GDNF protein (31.25 pg/mL–2.0 ng/mL).

### 2.7. Reverse Transcription and Quantitative PCR (qPCR) Analysis

Total RNA was extracted from each sample using the RNeasy Mini Kit (Qiagen, Valencia, CA). DNaseI was used to eliminate the possibility of genomic DNA contamination. RNA concentration was measured at a wavelength of 260 nm (A260) for each sample, and the purity of isolated total RNA was determined by the A260/A280 ratio. Quantitative RT-PCR analyses were only performed on samples with A260/A280 ratios between 1.9 and 2.1. Two micrograms of total RNA in a 20 *μ*L reaction were used for reverse transcription using an Omniscript cDNA Synthesis Kit (Qiagen, Valencia, CA). A primer set for each gene (Table 1) was designed using the cat genome browser (http://lgd.abcc.ncifcrf.gov/cgi-bin/gbrowse/cat/) and the primers synthesized commercially (Invitrogen). 

Quantitative PCR was performed using an Applied Biosystems 7500 Fast Real-Time PCR Detection System (Applied Biosystems, Foster, CA). Triplicate wells were used for each gene. A total volume of 20 *μ*L per well containing 10 *μ*L of 2x Power SYBR Green PCR Master Mix (Applied Biosystems, Foster, CA), 2 *μ*L of cDNA and gene-specific primers were used. Cycling parameters for qPCR were as following: the initial denaturation was at 95°C for 10 minutes, followed by 40 cycles of 15 s at 95°C and 1 minute at 60°C. To normalize template input, *β*-actin was used as an endogenous control and transcript level measured for each plate. The relative expression of the gene of interest was then evaluated using 7500 Fast System Sequence Detection Software, Version 1.4. The value obtained for Ct represents the number of PCR cycles at which an increase in fluorescence signal (and therefore cDNA) can be detected above background and the increase is exponential for the particular gene. Data were expressed as fold change relative to untreated controls after normalizing to *β*-actin. Error bars displayed the calculated maximum and minimum standard errors to the mean expression level of the triplicates.

### 2.8. Differentiation of Transduced cNPCs In Vitro

Transduced cNPCs were differentiated in UM without added EGF or bFGF and containing 10% FBS (UM-FBS). Cells (0.2 million) in UM were seeded in T25 culture flasks and allowed to attach, then culture medium was aspirated and replaced with either UM-FBS for differentiation or fresh UM for comparison. Conditioned media were collected and replaced with fresh media every 24 hours for 4 days and frozen for ELISA analysis. At the end of day 4, cells were trypsinized, counted, and ELISA analysis was performed on lysates as well as thawed media samples. For FACS analysis, transduced cNPCs were cultured in either UM-FBS or UM for 10 days prior to processing.

### 2.9. Immunocytochemistry

Transduced and nontransduced cNPCs were seeded in 4-well chamber slides (Nalge Nunc International, Rochester, NY) and allowed to grow for 3–5 days. Cells were re-fed every 2 days and fixed with freshly prepared 4% paraformaldehyde (Invitrogen) in 0.1 M phosphate-buffered saline (PBS) for 20 minutes at room temperature and washed with PBS. Cells were then incubated in antibody blocking buffer consisting of PBS containing 10% (v/v) normal goat serum (NGS) (Biosource, Camarillo, CA), 0.3% Triton X-100, 0.1% NaN3 (Sigma-Aldrich, Saint Louis, MO) for 1 hour at room temperature. Slides were incubated in primary antibodies (Table 2) overnight at 4°C. After washing the next morning, slides were incubated in fluorescent-conjugated secondary antibody (Alexa Fluor546 goat anti-mouse or goat anti-rabbit, 1 : 800 in PBS, BD) for 1 hour at room temperature. After washing, DAPI-containing Vectashield Hard Set Mounting Medium (Vector Laboratories, Burlingame, CA) was used to mount the slides for 20 minutes at room temperature. Negative controls for immunolabeling were performed in parallel using the same protocol but without primary antibody. Fluorescent staining was judged as positive only with reference to the negative controls. Immunoreactive cells were visualized and imaged using a fluorescent microscope (Eclipse E600, Nikon, Melville, NY).

## 3. Results

### 3.1. Transduction of Proliferating cNPCs by FIV-Based Vector

**Figure 1:**
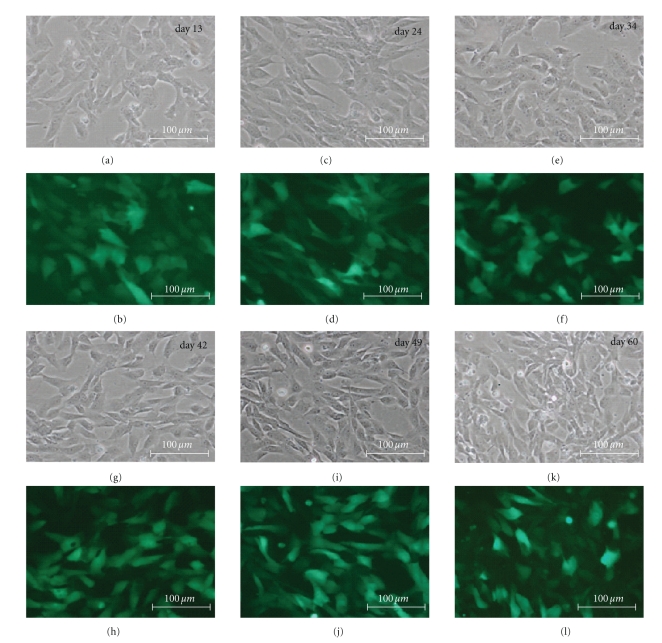
GDNF-transduced cNPCs: morphology and reporter gene expression. Feline NPCs transduced using a bicistronic lenti-GDNF-GFP vector and cultured under proliferation conditions (UM) for 60 days (p9–p26). Cellular growth, morphology, and GFP expression were monitored over this time period. In this figure, paired phase contrast ((a), (c), (e), (g), (i), (k)) and fluorescence ((b), (d), (f), (h), (j), (l)) micrographs of the same field are presented for each of 6 sequential time points, as indicated. Transduced cNPCs exhibited consistent mophologies, continued growth, and sustained GFP expression throughout the period examined. Bars = 100 *μ*m.

Currently, there are relatively few molecular tools with enhanced specificity for feline cells. Recent development of feline immunodeficiency virus- (FIV-) based vectors could present a means for improved delivery of transgenes into cells of this species. Here, we employed an FIV-based bicistronic vector for delivery of glial cell line-derived neurotrophic factor (GDNF) to cat neural progenitor cells (cNPCs). Forty eight hours after lenti-GDNF-GFP viral vector transduction, approximately 50% of cNPCs expressed the GFP reporter gene based on direct observation via fluorescence microscopy. To enrich for transgene-expressing cells, cNPCs were trypsinized at 72 hours postviral vector incubation and sorted by FACS based on GFP expression. The GFP-enriched population was subsequently cultured in Ultraculture-based proliferation medium (UM) for more than 60 days. High levels of GFP expression were sustained throughout this time period (Figure 1).

### 3.2. Expression of the GDNF Transgene Did Not Abrogate cNPC Proliferation

**Figure 2:**
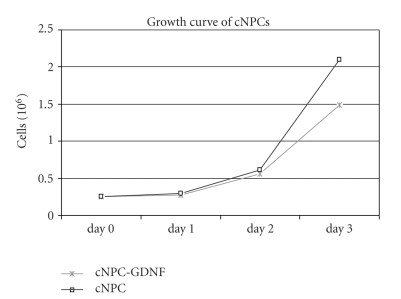
Growth properties of transduced versus nontransduced cNPCs. The growth of lenti-GDNF-GFP vector transduced cNPCs was compared to nontransduced cNPCs under proliferation conditions (UM). One flask of each type of cells was harvested and counted daily for 3 consecutive days. From this data it can be seen that the transduced cNPCs continued to proliferate despite overexpression of GDNF and that growth was similar to that of nontransduced cells out to day 2, after which the nontransduced cells exhibited relatively greater growth at the day 3 time point.

GDNF is known to have a range of biological activities in the context of the nervous system and cultured neural cell populations. Because this activity might extend to neural progenitors, we examined the effect of GDNF transduction on cNPC behavior, specifically the ability to proliferate. Proliferation is an important consideration for large-scale expansion of modified donor cell populations for use in transplantation studies. Transduced cNPCs continued to proliferate in a logarithmic manner, similar to but slightly slower than the nontransduced cNPCs (Figure 2). Conversely, the transduced cNPCs appeared to be somewhat more uniform, with less clumping and fewer floating cells, particularly when cells were cultured for more than 3 days in the same flask.

### 3.3. Transgene Expression Was Maintained under Differentiation Conditions

**Figure 3:**
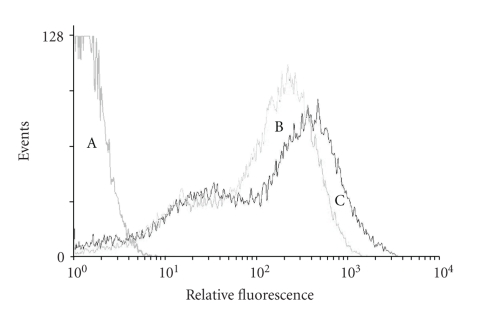
Flow cytometric analysis of GFP expression after induction of differentiation. Nontransduced cNPCs and lenti-GDNF-GFP vector transduced cNPCs cultured under proliferation conditions (UM) were compared to transduced cNPCs cultured for 10 days in Ultraculture-based medium without EGF or bFGF and containing 10% FBS in order to induce differentiation (UM-FBS). Curve A: nontransduced cNPCs as negative controls; curve B: lenti-GDNF-GFP transduced cNPCs and curve C: lenti-GDNF-GFP transduced cNPCs in UM-FBS. Induction of differentiation did not attenuate expression of the GFP reporter gene.

Neuronal differentiation has been implicated in gene silencing; therefore FACS analysis was performed to evaluate the effects of cell differentiation on GDNF transgene expression using the GFP reporter. Approximately 95% of transduced cNPCs expressed GFP, either when cultured in UM (proliferation conditions) or 10% FBS-containing UM (differentiation conditions). Among the cells expressing GFP, approximately 70% expressed GFP at high levels. There was no evidence of diminished GFP expression by the cells grown in the presence of FBS, thereby demonstrating maintained transgene expression was under differentiation conditions (Figure 3).

### 3.4. Transduced cNPCs Produced and Secreted Elevated Levels of GDNF

**Figure 4:**
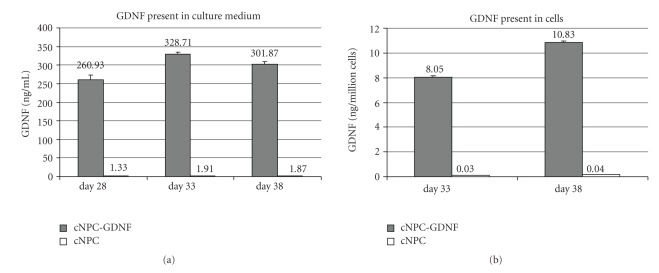
ELISA analysis of GDNF production by transduced cNPCs. (a) Lenti-GDNF-GFP vector transduced and nontransduced cells at passage 17 (cNPCp17) were seeded equally, under identical conditions, and allowed to grow for 15 days in UM, over which period the cells were passaged 3 times. At the time of each passage, culture media conditioned over the prior 48 hours was collected for ELISA assay. The conditioned media from transduced cNPCs was substantially enriched for GDNF compared to nontransduced cells. (b) Lenti-GDNF-GFP transduced and nontransduced cNPCp17 cells were trypsinized, lysed, and subjected to ELISA. GDNF was markedly elevated in lysates of transduced cells.

The levels of GDNF produced by transduced cNPCs, as present in conditioned culture medium and collected cell lysates, were analyzed by ELISA and compared to nontransduced controls. High levels of secreted GDNF were present in the culture medium of transduced cNPCs, measured on days 28, 33, and 38 posttransduction (Figure 4(a)). In addition, GDNF expression levels were considerably elevated in cell lysates extracted from transduced cultures on days 33 and 38 post-transduction (Figure 4(b)). Hence, transduced cNPCs continued to produce elevated levels of GDNF over a sustained period of time.

### 3.5. GDNF Expression Was Maintained under Differentiation Conditions

**Figure 5:**
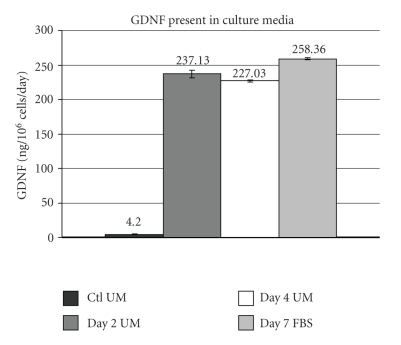
Effect of cell differentiation on transgene GDNF expression by ELISA. Lenti-GDNF-GFP vector transduced cNPCp24 cells were seeded equally in either UM (proliferation conditions) or Ultraculture-based medium without additional growth factors but containing 10% FBS (differentiation conditions, UM-FBS). Cultures were fed 24 hours prior to collecting GDNF conditioned media for ELISA assay at which time the cells were counted. ELISA data is presented as GDNF (ng) per million cells per day in order to further evaluate whether differentiation of transduced cNPCs had an influence on transgene expression. These data are consistent with sustained GDNF overexpression, confirming the flow cytometric data (Figure 3) that showed no evidence of diminished reporter gene expression in the UM-FBS treated population of transduced cNPCs.

Having shown above that expression of the GFP reporter was sustained when transduced cNPCs were subjected to differentiation conditions, and that the transduced cells overexpress GDNF, we next verified that GDNF expression was sustained during cNPC differentiation (Figure 5). Transduced cNPCs were cultured in UM without added growth factors and containing 10% FBS to induce cell differentiation and media were collected for ELISA. The level of GDNF produced under differentiation conditions was not diminished relative to proliferation conditions.

### 3.6. Effect of GDNF Overexpression on Neural Differentiation

**Figure 6:**
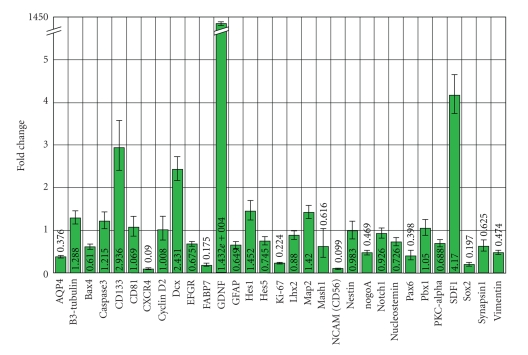
Expression profiles of cNPCs before and after transduction. The relative impact of GDNF overexpression on transcript expression levels was evaluated using qPCR analyses for a profile of 32 genes, which included *β*-actin as a housekeeping gene. Lenti-GDNF-GFP vector transduced cNPCp20 cells were compared to nontransduced cNPCp20 cells (with nontransduced cells set to 1.00). GDNF transcript level was over 14,000-fold higher in transduced versus nontransduced cells (note that *Y*-axis has break to accommodate value). The value for GDNF was vastly greater than any other changes in transcript level across the profile examined.

Neural progenitor cells have shown great promise as a source of neural cell types in transplantation studies. We therefore investigated whether genetically modified cNPCs retained their neural progenitor phenotype in the presence of high levels of GDNF expression, as assessed by a gene expression profile (Figure 6). qPCR analysis showed that transduced cNPC cells exhibited approximately 14,000-fold GDNF upregulation at the mRNA level compared to nontransduced controls. In transduced cells, expression levels of the progenitor cell markers nestin, vimentin, and sox2, as well as the neuronal marker *β*3-tubulin and the proliferation marker Ki-67 remained similar to that seen in nontransduced cells. Transduced cells also exhibited increased transcript levels for stromal cell-derived factor-1 (SDF1, 4.2-fold), prominin (CD133, 2.9-fold), doublecortin (DCX, 2.4-fold), and Hes1 (1.45-fold), as well as lower transcript levels for CXCR4, FABP7 and NCAM.

### 3.7. Examination of Protein Expression Using Immunocytochemistry

**Figure 7:**
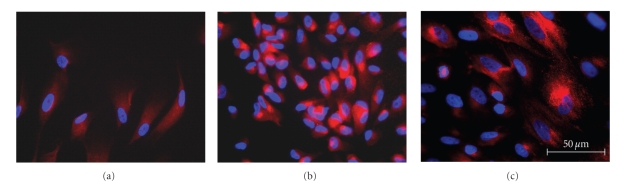
GDNF expression by cNPCs before and after transduction and differentiation. Immunocytochemistry (ICC) was performed on cNPCs using a rabbit anti-human GDNF antibody to evaluate expression of GDNF at the protein level, before and after transduction and before and after exposure to growth factor deprived/FBS-containing differentiation conditions (UM-FBS). (a) Nontransduced cNPCp20 cultured in UM (proliferation conditions) exhibit baseline cytoplasmic labeling for GDNF (red). (b) Lenti-GDNF-GFP vector transduced cNPCs cultured in UM show increased intensity of GDNF (red) labeling. (c) Transduced cNPCs cultured in UM-FBS (differentiation conditions) are larger in size and show persistent overexpression of GDNF (red), that is, heterogeneously distributed among the profiles. Nuclear labeling = DAPI (blue), scale bar = 50 *μ*m.

Immunocytochemical analysis demonstrated that cNPCs produced low levels of GDNF protein at baseline (Figure 7(a)), but that expression of the protein was substantially elevated following transduction with Lenti-GDNF-GFP (Figure 7(b)). To investigate the effect of differentiation on GDNF protein overexpression, cNPCs were cultured in either serum-free UM or UM containing 10% FBS for 5 days. Following the induction of differentiation, the cells appeared larger in size and GDNF expression was sustained, although heterogeneity of expression levels across the population was evident (Figure 7(c)).

**Figure 8:**
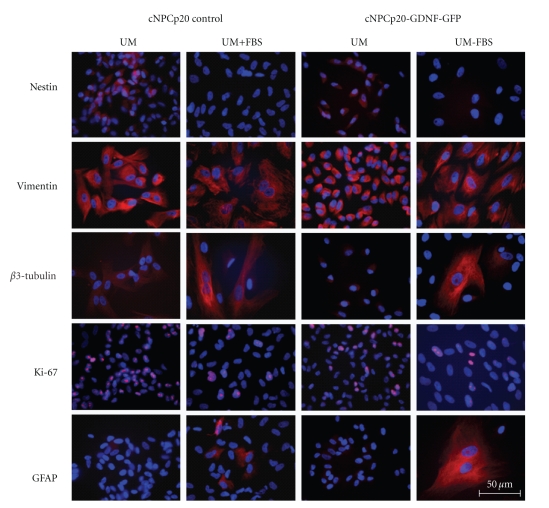
Expression of NPC and lineage markers before and after transduction and differentiation. The effects of passage number, induction of differentiation and GDNF transgene expression on the expression of 5 markers was evaluated using ICC. Nontransduced and lenti-GDNF-GFP vector transduced cNPCp20 were cultured in UM or UM-FBS, then immunolabeled with specific antibodies. The changes in expression patterns seen predominantly reflected exposure to differentiation conditions (alternating columns), with little that might be attributable to passage number or lenti-GDNF-GFP transduction. Scale bar = 50 *μ*m.

The expression of progenitor and lineage markers was also examined at the protein level, for both transduced and control cells, before and after induction of differentiation (Figure 8). The results verified the differentiating influence of the FBS-containing condition as follows. The neural progenitor cell marker nestin was only detected in cells grown in UM and was not seen in UM-FBS. Likewise, vimentin expression also decreased upon differentiation, although for this less-specific marker expression remained substantial. In contrast, *β*-tubulin III immunoreactivity was strikingly up-regulated in a subset of cells grown in UM-FBS, suggesting the induction of neuronal lineage. The proliferation marker Ki-67 was clearly downregulated in UM-FBS cultured cNPCs, whereas the glial marker GFAP was not detected under proliferation conditions, but was strongly up-regulated by a subset of cells cultured in UM-FBS. Having confirmed the differentiating influence of the UM-FBS conditions, the same immunocytochemical analysis was repeated on cNPCs of identical age that had been transduced using the lenti-GDNF-GFP vector. The results were equivalent, suggesting that the differentiation of cNPCs was not adversely influenced by transduction with GDNF (Figure 8).

## 4. Discussion

Among mammals, the highly developed visual system of the domestic cat has been studied in particular detail, owing in part to greater similarities with the human visual system as compared to laboratory rodents. This body of work, combined with the availability of naturally occurring retinal dystrophic mutants, would serve to recommend the cat as a powerful model for retinal regeneration research. A major limiting factor to regenerative research in this species is the paucity of available donor cells of the type suitable for such work, including stem, progenitor, or precursor cells of allogeneic origin. Furthermore, the use of these cells in transplantation studies would benefit from the inclusion of a reporter gene and, in some cases, additional transgenes of potential therapeutic value. 

Here we demonstrate the feasibility of using feline lentiviral vectors to genetically modify cNPCs for sustained delivery of GDNF. These cells possess multiple desirable features for use in transplantation studies including ease of expansion in vitro, coexpression of a green fluorescence protein (GFP) reporter gene serving to both confirm GDNF expression as well as allowing easy tracking of donor cells after transplantation, and sustained transgene expression following differentiation. In addition, they are allogeneic with respect to the targeted host species and therefore likely to be well tolerated without for the need of exogenous immune suppression [31]. 

The ability of a progenitor cell to sustain proliferation is important in order to avoid the necessity of repeated rederivation of the modified cell type. Importantly, the GDNF-GFP overexpressing cNPCs continued to exhibit log growth characteristics, indicating that neither the genetic modification process nor GDNF overexpression presents a major barrier to continued proliferation of these cells. Nevertheless, the growth of the GDNF-transduced cNPCs was less rapid than that of unmodified controls. This slower growth rate is also reflected in the lower number of cells that were Ki-67 positive following introduction of the transgene construct. Since we have recently shown that exogenous GDNF tends to promote, rather than hinder, the growth of murine RPCs [32], it seems unlikely that a feedback signaling mechanism involving the overexpressed cytokine would explain the behavior seen here. Perhaps the particularly high levels of transgene expression maintained by the GDNF-GFP transduced cNPCs results in a metabolic load that slows growth relative to unmodified cells. Alternatively, genetic modification could introduce abnormalities to the host genome, for instance as a function of the sites of transgene integration. 

Another consideration in terms of clinical application of transduced cells is the regulation of transgene expression. Sustained overexpression might result in undesired effects such as decreased sensitivity to the gene product, as might result from down-regulation of the corresponding growth factor receptor or, alternatively, toxic responses to high levels of the cytokine, either within the eye or systemically. Titrating the dose of transplanted cells should set an upper limit on GDNF delivery, since the progenitor cells tend to cease proliferation in vivo, however, a more sophisticated approach would be the use of inducible promoters which allow for the dynamic regulation of transgene expression levels.

Looking forward, the GDNF-GFP overexpressing cNPCs developed here are suitable for allogeneic transplantation to the vitreous cavity or subretinal space of cats with retinal disease. Of particular interest is the application of these cells to existing animals with photoreceptor dystrophy, such as the Swedish Abyssinian breed with the CEP290 mutation [29], with the goal of ameliorating visual loss through the sustained intraocular delivery of a neurotrophic factor. In vivo experiments in this nonrodent species would more realistically model the prospective treatment of analogous human conditions and could yield valuable information pertaining to the mechanisms of graft-mediated effects on host visual function.

## Figures and Tables

**Table 1:** Cat-specific primers for quantitative RT-PCR (GDNF = human).

Gene	Forward primer (5′-3′)	Reverse primer (5′-3′)
*β*-actin	GCCGTCTTCCCTTCCATC	CTTCTCCATGTCGTCCCAGT
Nestin	CTGGAGCAGGAGAAGGAGAG	GAAGCTGAGGGAAGCCTTG
Sox2	ACCAGCTCGCAGACCTACAT	TGGAGTGGGAGGAAGAGGTA
Vimentin	ATCCAGGAGCTACAGGCTCA	GGACCTGTCTCCGGTACTCA
Pax6	AGGAGGGGGAGAGAATACCA	CTTTCTCGGGCAAACACATC
Hes1	GCCAGCAGATATAATGGGAGA	GCATCCAAAATCAGTGTTTTCA
Hes5	CTCAGCCCCAAAGAGAAGAA	AGGTAGCTGACGGCCATCTC
Notch1	CAGTGTCTGCAGGGCTACAC	CTCGCACAGAAACTCGTTGA
Mash1	CATCTCCCCCAACTACTCCA	CCAACATCGCTGACAAGAAA
Ki-67	TCGTCTGAAGCCGGAGTTAT	TCTTCTTTTCCCGATGGTTG
DCX	GGCTGACCTGACTCGATCTC	GCTTTCATATTGGCGGATGT
*β*3-tubulin	CATTCTCGTGGACCTTGAGC	GCAGTCGCAATTCTCACATT
Map2	ACCTAAGCCATGTGACATCCA	CTCCAGGTACATGGTGAGCA
PKC-alpha	TTCACAAGAGGTGCCATGAA	CCATACAGCAATGACCCACA
GFAP	CGGTTTTTGAGGAAGATCCA	TTGGACCGATACCACTCCTC
Lhx2	GATCTGGCGGCCTACAAC	AGGACCCGTTTGGTGAGG
CD81	CCACAGACCACCAACCTTCT	CAGGCACTGGGACTCCTG
CD133	AGGAAGTGCTTTGCGGTCT	TGCCAGTTTCCGAGTCTTTT
NCAM (CD56)	AGAACAAGGCTGGAGAGCAG	TTTCGGGTAGAAGTCCTCCA
EGFR	AACTGTGAGGTGGTCCTTGG	CGCAGTCCGGTTTTATTTGT
NagoA	TTTGCAGTGTTGATGTGGGTA	TAACAGGAACGCTGAAGAGTGA
SDF1	ACAGATGTCCTTGCCGATTC	CCACTTCAATTTCGGGTCAA
CXCR4	TCTGTGGCAGACCTCCTCTT	TTTCAGCCAACAGCTTCCTT
Cyclin D2	CAAGATCACCAACACGGATG	ATATCCCGCACGTCTGTAGG
Pbx1	CTCCGATTACAGAGCCAAGC	GCTGACCATACGCTCGATCT
FABP7	TGGAGGCTTTCTGTGCTACC	TGCTTTGTGTCCTGATCACC
AQP4	TACACTGGTGCCAGCATGA	CACCAGCGAGGACAGCTC
Nucleostemin	CAGTGGTGTTCAGAGCCTCA	CCGAATGGCTTTGCTGTAA
Synapsin1	ACGACGTACCCTGTGGTTGT	CGTCATATTTGGCGTCAATG
Caspase 3	ATGGAGAACAGTGAAAACTCAGTGG	AATTATTATACATAAACCCATTTCAGG
Bax 4	CTGAGCAGATCATGAAGACAGG	GTCCAGTTCATCTCCGATGC
hGDNF*	TGGGCTATGAAACCAAGGAG	CAACATGCCTGCCCTACTTT

*Human GDNF gene.

**Table 2:** Primary antibodies used for immunocytochemistry on cNPCs.

Target	Antibody type	Reactivity in retina	Source	Dilution
Nestin	Mouse monoclonal	Progenitors, reactive glia	BD	1 : 200
Vimentin	Mouse monoclonal	Progenitors, reactive glia	Sigma	1 : 200
Ki-67	Mouse monoclonal	Proliferating cells	BD	1 : 200
GFAP	Mouse monoclonal	Astrocytes, reactive glia	Chemicon	1 : 200
*β*3-tubulin	Mouse monoclonal	Immature neurons	Chemicon	1 : 200
GDNF	Rabbit polyclonal	Growth factor	SCBT	1 : 200
